# Evaluating the Effects of a Self-Help Mobile Phone App on Worry and Rumination Experienced by Young Adults: Randomized Controlled Trial

**DOI:** 10.2196/51932

**Published:** 2024-08-13

**Authors:** Daniel Edge, Edward Watkins, Alexandra Newbold, Thomas Ehring, Mads Frost, Tabea Rosenkranz

**Affiliations:** 1 Mood Disorders Centre School of Psychology University of Exeter Exeter United Kingdom; 2 Department of Psychology Ludwig-Maximilians-University of Munich Munich Germany; 3 Monsenso A/S Copenhagen Denmark

**Keywords:** worry, rumination, repetitive negative thinking, prevention-mechanism, well-being, depression, anxiety, mobile-based interventions, mobile phone, mobile health application, app, application

## Abstract

**Background:**

Delivery of preventative interventions via mobile phone apps offers an effective and accessible way to address the global priority of improving the mental health of adolescents and young adults. A proven risk factor for anxiety and depression is elevated worry and rumination, also known as repetitive negative thinking (RNT).

**Objective:**

This was a prevention mechanism trial that aimed to investigate whether an RNT-targeting self-help mobile phone app (MyMoodCoach) reduces worry and rumination in young adults residing in the United Kingdom. A secondary objective was to test whether the app reduces symptoms of anxiety and depression and improves well-being.

**Methods:**

A web-based, single-blind, 2-arm parallel-group randomized controlled trial was conducted with 236 people aged between 16 and 24 years, who self-reported high levels of worry or rumination. Eligible participants were randomized to an active intervention group (usual practice, plus up to 6 weeks of using the RNT-targeting mobile app, n=119) or a waitlist control group (usual practice with no access to the app until after 6 weeks, n=117). The primary outcome was changes in worry and rumination 6 weeks after randomization. Secondary outcomes included changes in well-being and symptoms of anxiety and depression after 6 weeks and changes in all measures after 12 weeks.

**Results:**

Participants randomly allocated to use the RNT-targeting self-help app showed significantly lower levels of rumination (mean difference –2.92, 95% CI –5.57 to –0.28; *P*=.03; η_p_^2^=0.02) and worry (mean difference –3.97, 95% CI –6.21 to –1.73; *P*<.001; η_p_^2^=0.06) at 6-week follow-up, relative to the waitlist control. Similar differences were observed for well-being (*P*<.001), anxiety (*P*=.03), and depression (*P*=.04). The waitlist control group also showed improvement when given access to the app after 6 weeks. Improvements observed in the intervention group after 6 weeks of using the app were maintained at the 12-week follow-up point.

**Conclusions:**

The MyMoodCoach app had a significant positive effect on worry and rumination, well-being, anxiety, and depression in young adults, relative to waitlist controls, providing proof-of-principle that an unguided self-help app can effectively reduce RNT. This app, therefore, has potential for the prevention of anxiety and depression although longer-term effects on incidence need to be directly evaluated.

**Trial Registration:**

ClinicalTrials.gov NCT04950257; https://www.clinicaltrials.gov/ct2/show/NCT04950257

**International Registered Report Identifier (IRRID):**

RR2-10.1186/s12888-021-03536-0

## Introduction

There is growing concern about the early onset of mental health disorders in adolescents and young adults [1]. Poor mental health during adolescence can severely affect a young person’s future life chances and can have a significant negative impact on health, education, and employment in later life [2,3]. Depression and anxiety are 2 of the most common mental health problems in adolescents and young adults, which significantly contribute toward global disability and produce a high economic burden [4]. In 2020, the prevalence of depression and anxiety disorders in adolescents globally was estimated at between 25% and 31% [5].

Although effective treatments for depression and anxiety have been developed and delivered in many countries, the evidence suggests that the overall prevalence of both disorders remains largely unchanged [6]. Because the prevalence of these common mental health disorders is so high, it is not possible for traditional delivery models of psychological intervention, such as a course of face-to-face sessions, to fully address the global need for treatment as it is unlikely there will ever be a sufficient number of trained mental health professionals [7]. Further, even if acute treatments were made widely available, as a consequence of the high levels of recurrence and relapse for depression and anxiety, it has been estimated that only a partial reduction of the overall prevalence could be achieved [6,8]. Effective prevention approaches are, therefore, considered essential in lowering overall prevalence rates as they have the potential to reduce both initial and recurrent episodes, thereby decreasing the demand for acute treatment [9]. Given the substantial increase in the incidence of depression and anxiety during mid-adolescence peaking in young adulthood [2], adolescents and young adults are considered an important group for which prevention of poor mental health is urgently needed [10-12].

While prevention interventions for anxiety and depression already exist for adolescents and young people, systematic reviews suggest that effect sizes are relatively small [13-15]. Further, most prevention interventions are delivered in person and require considerable input from trained professionals, such as teachers and therapists, which can increase costs and limit their availability [16]. While there is some evidence that supported interventions may have better adherence and efficacy than unguided interventions, their coverage and volume of delivery are constrained because each individual supporting the intervention has a finite capacity, such that these interventions cannot be made available to all who may benefit. As such, to ensure the large-scale coverage needed for effective mental health prevention approaches, there is potential value in supplementing supported interventions with ones that can be delivered to multiple users simultaneously and do not require additional input from practitioners (ie, nonconsumable self-help interventions [17]). The use of such nonconsumable self-help interventions would substantively increase the scalability and availability of preventative interventions making them more suitable for use as a public health approach at a population level.

The use of the internet and mobile phones has been increasingly explored as an avenue that may be able to increase the accessibility of prevention approaches and reduce the cost of intervention [18]. While internet and mobile-based interventions have many similarities, their method of delivery can vary. Mobile-based interventions, for example, are typically delivered through dedicated mobile apps and accessed on mobile phones, whereas internet-based interventions use web-based (or compatible) platforms designed to be accessed via laptops or personal computers. Internet-based interventions tend to deliver therapeutic content made up of pages working through a specific topic and more closely resemble a structured therapeutic session, for example, taking a set amount of time to complete and working through set content. Mobile apps, however, are based on more flexible use of “bite-sized” information and exercises, with less information on the screen, and allowing the user to “swipe” through available material to access the content they desire. The use of mobile phones is near ubiquitous in young people in the United Kingdom with an estimated 99% of those aged between 16 and 24 years using a smartphone on a regular basis in 2023 [19]. Further, mobile apps can help integrate behavioral changes into daily life—the app is always available to the user, making it well-suited for changing unhelpful habits. Mobile apps, therefore, provide a unique avenue in which to engage young adults in health-related activities, as well as promote good mental health and prevention strategies [20,21]. Despite a huge increase in the number of mobile-based mental health apps over the last 10 years [22], only a small number have been developed with scientific rigor. Further, many do not use established treatment principles nor have they been rigorously tested in robust well-powered randomized controlled trials (RCTs) [23]. While emerging evidence suggests that mobile-based apps can deliver efficacious treatment interventions for anxiety and depression [24,25], there have not been many trials examining their use for well-being promotion and prevention of poor mental health in young people specifically [26,27].

A psychological process thought to be key when promoting good well-being and preventing mental health problems is repetitive negative thinking (RNT) [28]. RNT is defined as a pattern of thinking that is repetitive, difficult to manage, and focused on negative content, which can significantly impact an individual’s well-being and emotional functioning [28-30]. RNT encompasses various thought patterns, but the exemplars of RNT are worry and rumination which have been identified as robust risk factors for several mental health disorders [31]. Worry is described as a relatively uncontrollable chain of negative thinking about the future in the form of “What if” type questions. Such thoughts can focus on typical everyday activities (such as work and relationships), as well as more catastrophic concerns (such as worrying that you may get hit by a falling tree) [32]. Rumination is a form of dysfunctional, negative thinking, which focuses on analyzing the causes and consequences of negative events. This can involve dwelling on past events and continually going over and over why things went wrong [32]. The degree of rumination experienced by an individual has been shown to predict the onset and duration of major depressive episodes [33,34], as well as the severity of depressive symptoms [35,36]. Rumination has also been shown to mediate the effects of other identified risk factors, such as neuroticism and stressful life events, on the onset of depressive episodes [37]. Worry has been identified as having strong associations with symptoms of depression and anxiety [38]. There is also evidence that increased levels of worry are predictive of greater symptom severity for both anxiety and depression [39] and that daily worrying predicts subsequent increases in anxiety [40]. There is extensive evidence that worry and rumination share a common process including evidence that they are highly correlated with each other and that this common factor accounts for their relationship with anxiety and depression, which underpins the conceptualization of the wider process of RNT [28,29]. There is robust evidence for RNT as a transdiagnostic risk factor, which is common across many psychological mood disorders including depression and anxiety [32,41]. The induction of RNT has been found to exacerbate symptoms of anxiety and depression such as negative thinking, delayed decision-making speed, poor problem-solving, and negative affect [42-45]. There is extensive evidence that RNT predicts future levels of depressive and anxiety-related symptoms, as well as the onset of depression [28,46]. As rumination and worry have been shown to be proximal risk factors that affect onset, maintenance, and relapse, RNT is considered an underlying mechanism for both anxiety and depression and is identified as an important target for preventative interventions [41,47].

There are several interventions that have been designed specifically to reduce RNT and improve general mental well-being, including those which have been trialed as digital interventions delivered via the internet. An RCT of a preventative intervention designed to target excessive levels of RNT found that both a group-based and internet version of a rumination-focused Cognitive Behavioral Therapy program significantly reduced RNT (*d*=0.53-0.89) and symptom levels of anxiety and depression (*d*=0.36-0.72) compared to a waitlist group [48]. Another trial involving 235 high-risk participants found that an internet-based version of the program reduced the risk of depression by 34% relative to controls and that participants showed a significant improvement in RNT and depressive symptoms in the short to medium term [49]. While there is promising evidence, therefore, that preventative interventions focused on reducing RNT are effective when delivered via the internet, there is limited research exploring the efficacy of RNT-targeting interventions delivered via a mobile phone app.

The Assessing and Enhancing Emotional Competence for Well-being in the Young project (ECoWeB) aimed to address the gap in large-scale trial evidence for preventative apps by developing and evaluating a self-guided mobile phone app (MyMoodCoach). The app for this large-scale trial was designed to promote emotional well-being and prevent mental health problems in adolescents and young adults through engaging and personalized tools that train psychological skills [50]. Specifically, this app was personalized by targeting the 2 most problematic components of emotional competence skills (from a set of 4: emotional regulation, emotional appraisal achievement context, emotional appraisal social context, and emotional perception and knowledge) for the user, which were identified in their baseline assessments [50]. One of these components was addressed via a module focused on targeting RNT for individuals identified as having elevated levels of RNT at baseline (reflecting poor emotional regulation and emotional competence skills). The trial described in the current paper is an off-shoot study of this larger project, specifically focused on testing the value of an app that targets RNT. In this study, we tested a standalone variant of the app used in the large-scale trial that included only the module focused on targeting RNT, aiming to shift users toward more adaptive emotional regulation skills.

Developing trials to evaluate preventative interventions can take considerable time and resources. However, while outcomes for prevention interventions typically focus on reducing the incidence (ie, new cases) of a specific mental health problem, prevention mechanism trials concentrate on the underlying etiological processes. This allows for a more detailed examination of proximal mechanisms. Prevention mechanism trials, therefore, offer an efficient way of establishing whether interventions can reduce risk factors associated with pathology, thereby evaluating their potential for prevention [51]. Since worry and rumination are established risk factors for anxiety and depression, this trial was a prevention mechanism trial to test whether the use of an app targeting RNT can reduce worry and rumination in young people, thus evaluating its potential as a prevention intervention. Our primary hypothesis was that people allocated to the RNT-targeting mobile app would show significantly lower levels of rumination and worry relative to those allocated to the waitlist control arm. A second hypothesis was that those allocated to the mobile app would also report significantly lower symptoms of anxiety and depression and higher well-being relative to waitlist control.

## Methods

### Study Design

A superiority 2-arm parallel-group RCT was conducted comparing an active intervention arm (usual practice plus up to 6 weeks of using the RNT-targeting mobile app) with a waitlist control arm (usual practice with access to the app only after a 6-week wait). As it was not possible to blind participants to their allocated groups, this was a single-blind RCT with only the researcher blind to participant group allocation. Further details can be found in the trial protocol [52]. This study was conducted according to the CONSORT (Consolidated Standards of Reporting Trials) guidelines [53,54] and extensions for nonpharmacological treatment interventions, as well as CONSORT-EHEALTH (Consolidated Standards of Reporting Trials of Electronic and Mobile HEalth Applications and on Line Tele Health) guidelines for improving and standardizing evaluation reports of web-based and mobile health interventions (Multimedia Appendix 1) [55].

### Recruitment

We recruited young adults aged between 16 and 24 residing in the United Kingdom. The study was advertised on social media platforms, including Facebook (Meta Platforms) and Instagram (Meta Platforms), as well as an internal research recruitment system (Sona) used by the University of Exeter. Only participants who reported elevated levels of RNT at baseline were eligible for this trial. This was defined as scoring above the 50th percentile (ie, top half of scale) on either the Ruminative Response Scale (RRS; >34) or the Penn State Worry Questionnaire (PSWQ; >41). Participants also had to be aged between 16 and 24 years, currently residing in the United Kingdom, possess basic literacy in English and have access to a smartphone (either Android or iOS). Recruitment commenced on May 14, 2021, and ceased on October 11, 2021.

**Figure 1:**
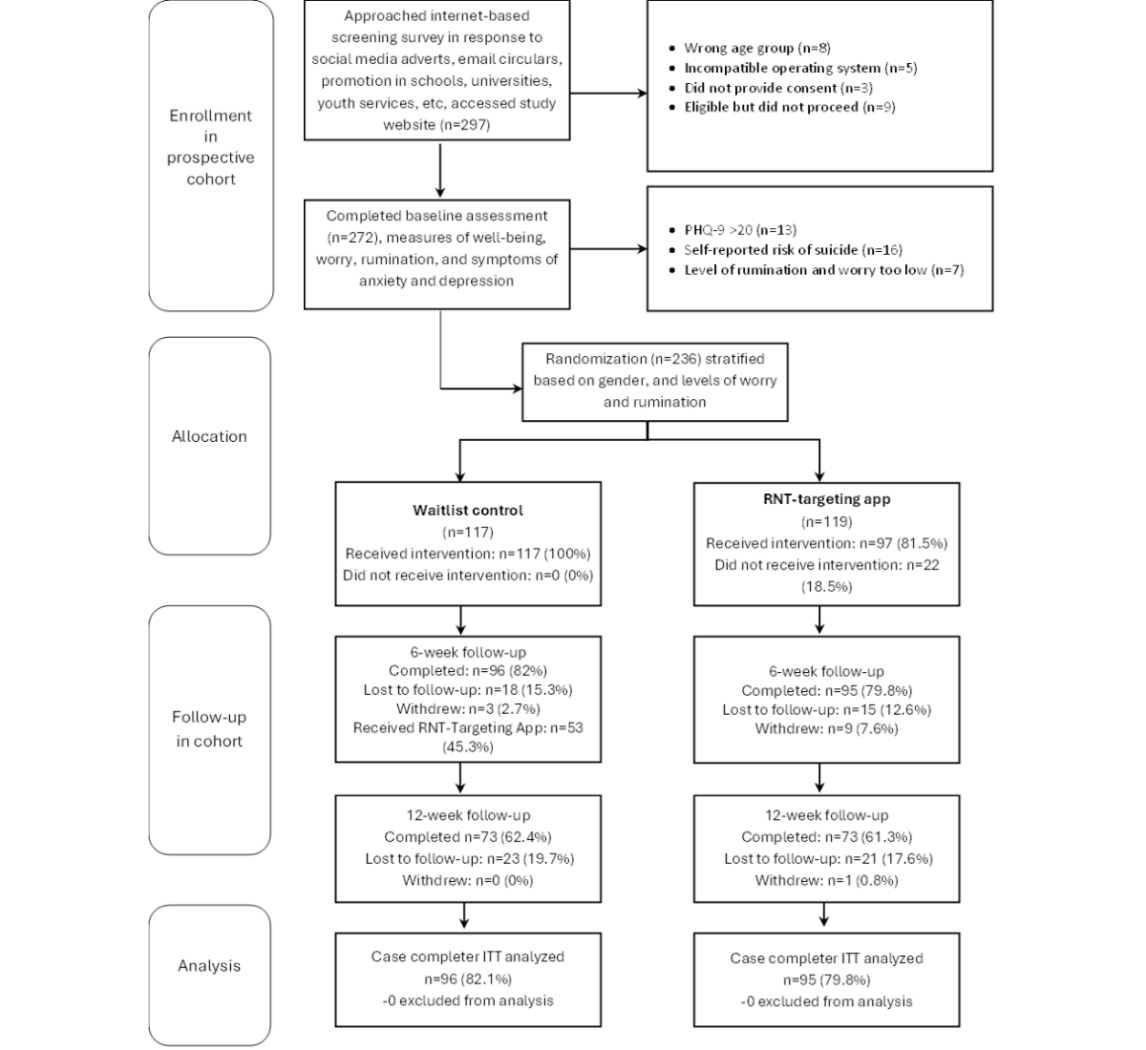
CONSORT flow diagram for the trial. CONSORT: Consolidated Standards of Reporting Trials; ITT: intention-to-treat; PHQ-9: Patient Health Questionnaire-9; RNT: repetitive negative thinking.

Participants were excluded at baseline if they reported elevated symptoms of depression indicating they required more specialist treatment, defined as having a score of 20 or higher on the Patient Health Questionnaire-9 (PHQ-9) [56] because this suggests a severe level of symptoms where a more intensive treatment from a mental health professional would be indicated. Participants who reported elevated symptoms of anxiety were included and automatically provided with additional information for accessing support if required. Other exclusion criteria included active suicidality or self-reported to be currently receiving treatment for a mental health problem (ie, psychological therapy, counseling, or psychiatric medication) at baseline. Those who self-reported having a current diagnosis of clinical depression, bipolar disorder, or psychosis were also excluded. A summary is given in the CONSORT flow diagram (Figure 1).

### Screening and Consent Procedure

Potential participants were directed to a website providing further information about the study and were prompted to answer some prescreening questions, regarding their age and current experiences of mental health. Individuals who were not suitable at this stage (eg, outside of the specified age range) were automatically directed to a web page explaining why they were not suitable for the trial. Participants who passed this prescreening stage were then provided with an information sheet and asked to consent to provide contact details and complete a baseline assessment where suitability for the trial (ie, level of RNT and symptoms for anxiety and depression) was assessed more thoroughly. Following the baseline assessment, participants who met all eligibility criteria were then asked to consent to take part in the trial before being randomized into one of the 2 arms. Everyone accessing the trial website was provided with contact details for the research team.

### Randomization

Randomization was conducted independently using a pregenerated computerized allocation algorithm. Randomization was in a 1:1 ratio and stratified across each arm according to the sex (male, female, and nonbinary) and level of RNT (50th-75th percentile vs 75th percentile or higher). Participants scoring in the highest quartile (at or above the 75th percentile) on either the RRS or PSWQ and in the top tercile on the other measure were identified as having high RNT, based on criteria used by Topper et al [48], which have previously been found to predict increased risk for subsequent depression [10].

### Participant Flow

Overall, 297 people completed the prescreening process of whom 272 went on to complete baseline questionnaires and have their suitability for the trial assessed more thoroughly. In total, 236 participants met the eligibility criteria and were randomized to either receive access to the digital RNT-targeting self-help app straight away (n=119) or after a period of 6 weeks (n=117). The rate of follow-up attrition was 19.07% (n=45; intervention arm, n=24; and waitlist control, n=21) at 6 weeks and 38.14% (n=90; intervention arm, n=46; and waitlist control, n=44) at 12 weeks. These calculations included 11 (4.6%) participants who contacted the research team requesting to withdraw from the study, 1 of who did so after completing their 6-week follow-up. There were no missing data from any of the participants who completed their surveys. Further details are given in the CONSORT flow diagram (Figure 1).

### Interventions

#### Digital RNT-Targeting Self-Help App (Intervention Arm)

The self-help app used in this trial (called MyMoodCoach) was a focused version of the app evaluated in the main ECoWeB trial [50]. This standalone version was not personalized to each user and was specifically focused on reducing RNT to help improve emotion regulation, that is, it only included the emotional regulation rumination-focused module. The app included self-monitoring, psychoeducation, and active self-help exercises based on RNT-specific strategies from an evidence-based, rumination-focused cognitive behavioral therapy program intervention [48,49,57-59], adapting these evidence-based interventions from an internet-delivery context to an app format. Core elements of the intervention were designed to break the ruminative habit and enable users to shift toward a more helpful processing style. This involved coaching participants to spot warning signs for rumination and worry, and then plan alternative strategies. These included being more active, slowing things down, breaking tasks down, opposite action, relaxation, concrete thinking, becoming absorbed, self-compassion, and assertiveness. Participants were prompted to practice alternative strategies in response to their warning signs. The user interface includes text, pictures, audio recordings, animations, audio exercises, and questionnaires with tailored automated feedback. There was also a self-monitoring component which prompted users 5 times a day to rate their mood and level of rumination. The app featured a menu structure including a dashboard to monitor notifications and progress, a library function that had psychoeducation and explanatory animated videos, and an explore function to graph the self-monitoring responses made by the participant. The app also included challenges that provide learning exercises (eg, behavioral exercises) and tools that are brief strategies that young people can use at the moment when they need them (for example, compassion and relaxation exercises). The app was entirely automated (ie, self-guided) and designed for use on both iOS and Android phones. The app was accessed for free via each participant’s smartphone app store.

**Figure 2:**
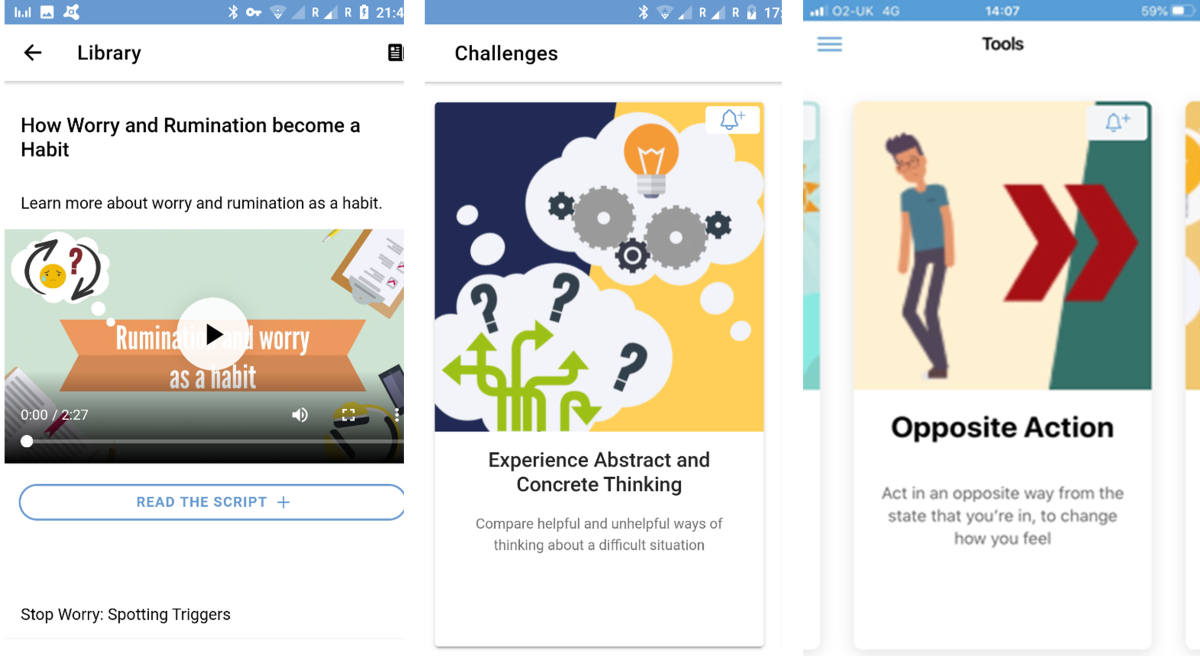
Screenshots from the MyMoodCoach app from left to right: screen from the library of visual resources, screen within the challenges menu to enter the abstract and concrete thinking challenge, screen within the tools menu to enter the opposite action tool.

Changes from the proven internet versions of the intervention [9,11] included information condensed so that “bite-sized” content reflecting 1 point of information was presented per swipe of the mobile phone screen and could be consumed in brief moments of time (rather than by scrolling down web pages each with multiple points of information); greater flexibility of use such that users could select any element of the app in any order from the menu (eg, use of tools or challenges) unlike the internet-delivered interventions, which tended to be more modular and arranged in a fixed structure and order, as a “lesson” or “session” that might take an hour to work through in its entirety. Some screenshots for the app are provided in Figure 2 and further details can be accessed via Multimedia Appendix 2.

#### Waitlist Control Group

The waitlist control group received access to the RNT-targeting digital self-help app after a 6-week wait.

### Baseline and Follow-Up Assessments

All outcome measures were completed by participants independently via a website at baseline, with follow-up assessments at 6-weeks and 12-weeks post randomization. Demographic information including, age, gender, employment status, and ethnicity, was collected only at baseline. Additional feedback about experiences of using the mobile app was only collected at the 12-week follow-up point. Automated emails were sent to participants at each follow-up point with a link to complete their survey. Further reminders were sent manually via email and text message if there was no response to the automated reminders.

As an incentive, all participants who completed their follow-up surveys were entered into a prize draw. Participants recruited via the University of Exeter Sona system could choose whether to be entered into the prize draw or receive course credits. Out of those eligible to participate in the trial, 82 (32.7%) chose to receive course credit. The researcher who was blind to treatment allocation was the only team member involved in sending out reminders to complete follow-up surveys. Other members of the team were available to follow up on issues of risk and answer technical queries from participants. Throughout the trial process, there were no deviations from the planned procedure with no incidents of unblinding being reported.

### Outcomes

The primary outcome for this trial was changes in the levels of rumination and worry at the primary end point (6 weeks after randomization). Rumination was measured using the RRS [36]. The measure consists of 22 items that assess the tendency for an individual to respond to depressed moods with a focus on either the self, depressive symptoms, or negative consequences. Items are scored from 1 (almost never) to 4 (almost always) and total scores can range from 22 to 88 with higher scores indicating a higher level of rumination. The RRS has good internal consistency in an adolescent population (α=.88) [60] and test-retest reliability ranges from moderate to high (*r*=0.47 for over 1 year and *r*=0.80 for over 5 months) [33,61].

Worry was measured using the PSWQ [62], a 16-item self-assessment that assesses the intensity, tendency, and uncontrollability of worrying thoughts using a 5-point Likert scale for each item. Items are scored from 1 (not at all typical of me) to 5 (very typical of me) and total scores can range from 16 to 80 with higher scores indicating greater levels of worry. The measure has high internal consistency in both clinical and nonclinical samples (α=.88-.95), as well as good test-retest reliability (*r*=0.72-0.93 for 2 weeks and 1 month, respectively) [62].

Secondary outcomes for this study included changes in mental well-being and symptoms of anxiety and depression at the primary end point, as well as changes in all measures between 6-week and 12-week follow-up. Mental well-being was measured using the Warwick-Edinburgh Mental Well-being Scale (WEMWBS) [63]. The WEMWBS has demonstrated high internal consistency in both student and general populations (α=.89-.91), with good test-retest reliability (intraclass correlation coefficient=0.83; *P*<.01) [63]. The Generalized Anxiety Disorder questionnaire (GAD-7) [64] was used to assess symptoms of anxiety. Symptoms of depression were assessed using the PHQ-9 [65]. Both measures have demonstrated good internal consistency (α=.86-.92), as well as good test-retest reliability (0.83-0.84) [66].

To assess user’s perspective on the app, participants were asked to rate the app on its ease of use, look and feel, features, and content on a 5-point Likert scale of 1 (terrible) to 5 (brilliant), as well as provide qualitative feedback about the positives and negatives of using the app. These questions were devised by the lead researcher and adapted from components of the technology acceptance model (TAM) and user experience (UX) design approaches [67,68].

### Statistical Analysis

#### Power

An estimated minimum clinically important difference (MCID) for the primary outcome of the RRS was used to calculate the sample size for this study. One recommended approach for identifying the MCID is half of the SD for the respective index [69]. A conservative estimate of the normative SD for the RRS was identified based on previous research (mean 47.19, SD 7.58 to mean 48.89, SD 8.51) [48]. A reduction of 4 points on the RRS, therefore, was identified as an appropriate MCID. Using an α of .05 with 90% power, for an MCID of 4 required 85 participants per group (170 in total). Allowing for a 20% follow-up attrition rate, we aimed to recruit a minimum of 204 participants (102 per arm).

#### Analysis Plan

Data were analyzed using SPSS (version 28; IBM Corp) and the statistical analytical plan was finalized prior to data being unblinded. The primary analysis was based on intention-to-treat and all participants, regardless of their level of engagement, were included [70]. Missing data were handled via multiple imputations (MI) using a linear regression model with a monotone imputation method. As multiple imputations had equivalent results to case-completer analyses, only the case-completer intention-to-treat analysis results have been reported. Analysis of covariance (ANCOVA) at 6 weeks was used for both primary and secondary outcomes, with baseline scores as the covariate. ANCOVA was also used for both primary and secondary outcomes at 12 weeks with 6-week follow-up scores as the covariate.

Feedback about user experience was collated and analyzed using NVivo software (Lumivero) which identified common words and phrases occurring within the data to generate preliminary codes. The same software was then used to automatically group the identified codes into suggested themes. These preliminary themes were then checked and refined by the primary researcher before being finalized.

### Ethical Considerations

Ethical approval for this study was obtained from the Ethics Committee of the School of Psychology, University of Exeter (eCLESPsy001977v5.1). All participants provided written informed consent. Data were collected in a pseudonymized manner and stored securely.

## Results

### Overview

Baseline demographics across both arms for the study are shown in Table 1. All participants were aged between 16 and 24 years and were predominantly White female students.

**Table 1:** Baseline demographics for each condition.

Baseline characteristics			RNT^a^-targeting app (n=119)		Waitlist control (n=117)
Age, mean (SD)			18.44 (2.01)		18.56 (2.4)
**Sex, n (%)**					
	Male	15 (12.6)		16 (13.7)	
	Female	109 (84)		98 (83.8)	
	Non-binary or third gender	4 (3.4)		3 (2.6)	
**Ethnicity, n (%)**					
	White	95 (79.8)		96 (82.1)	
	Black, African, Caribbean, or Black British	2 (1.7)		1 (0.9)	
	Asian or Asian British	14 (11.8)		15 (12.8)	
	Mixed or other ethnic group	8 (6.7)		5 (4.3)	
**Employment, n (%)**					
	Employed (full-time)	5 (4.2)		9 (7.7)	
	Employed (part-time)	11 (9.2)		14 (12.0)	
	Unemployed	8 (6.7)		4 (3.4)	
	Student	95 (79.8)		90 (76.9)	
**History of mental health problems, n (%)**					
	Yes	18 (15.1)		19 (16.2)	

^a^RNT: repetitive negative thinking.

### Outcome at Primary End Point (6 Weeks)

**Figure 3:**
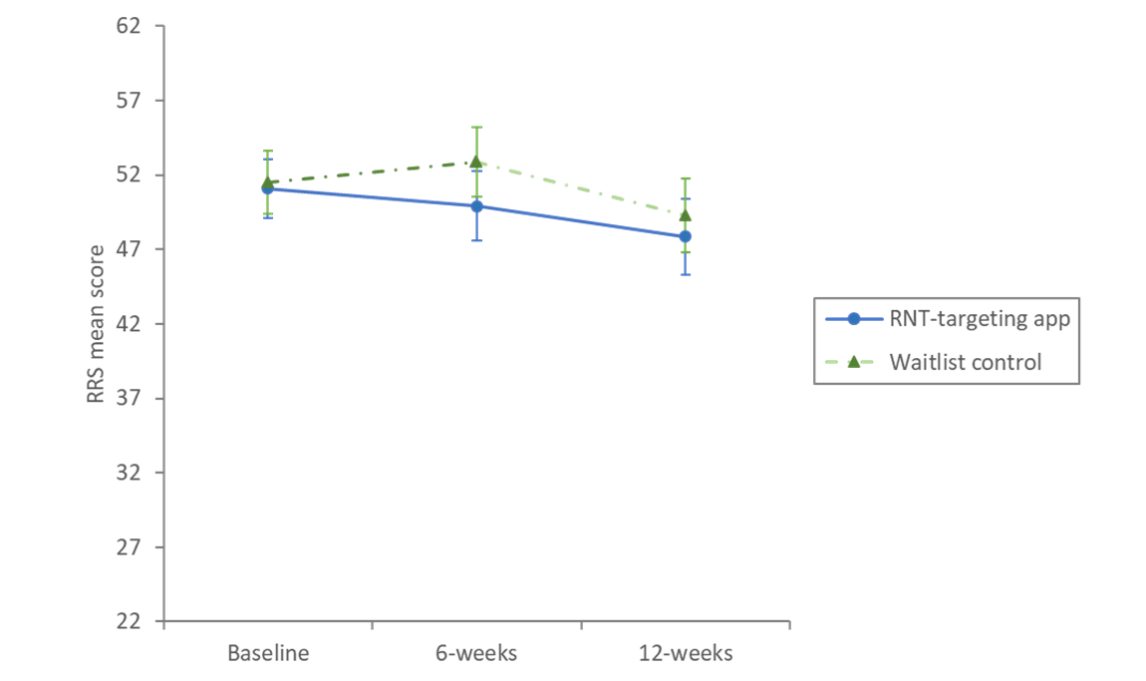
Graph of mean RRS scores for RNT-targeting app and waitlist control participants with 95% CI error bars. RNT: repetitive negative thinking; RRS: Ruminative Response Scale.

**Figure 4:**
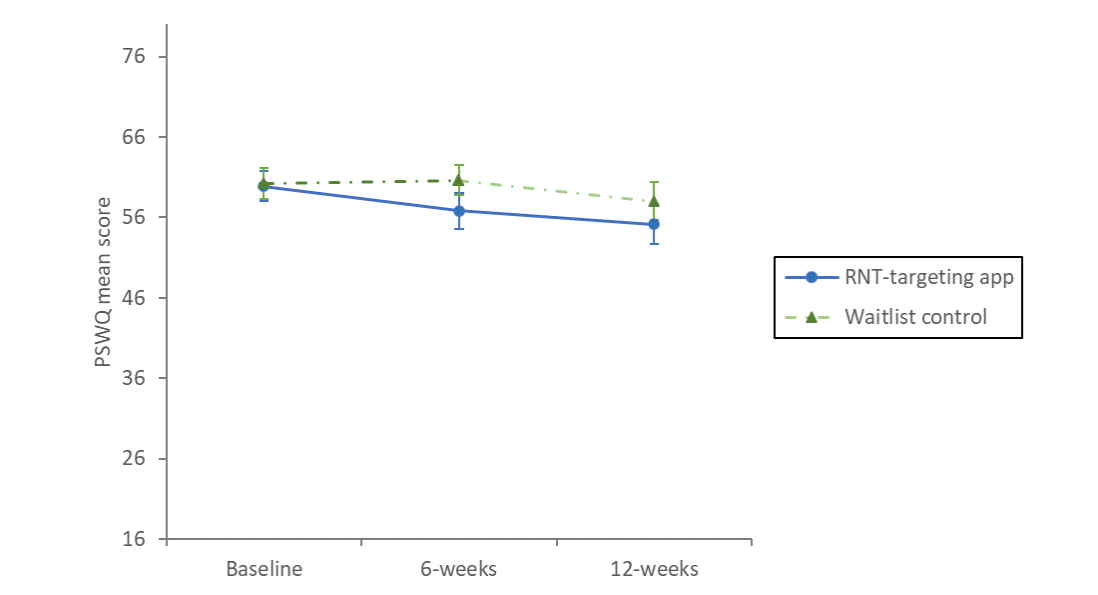
Graph of mean PSWQ scores for RNT-targeting app and waitlist control participants with 95% CI error bars. PSWQ: Penn State Worry Questionnaire; RNT: repetitive negative thinking.

Means and SDs for each measure and arm at each time point are shown in Table 2. One-way condition (intervention vs control) ANCOVAs found a significant main effect of intervention condition on rumination (Figures 3 and 4; *F*_1,188_=4.72; *P*=.03; η_p_^2^=0.02), worry (*F*_1,188_=12.24; *P*<.001; η_p_^2^=0.06), depression (*F*_1,188_=4.14; *P*=.04; η_p_^2^=0.02), and anxiety (*F*_1,188_=5.43; *P*=.02; η_p_^2^=0.03). These significant effects reflect that after adjusting for the covariate of baseline scores, participants randomized to use the RNT-targeting app in the intervention arm reported significantly lower rumination, worry, depression, and anxiety at 6-week follow-up than those randomized to the waitlist control (mean difference [rumination]: –2.92, 95% CI –5.57 to –0.28; mean difference [worry]: –3.97, 95% CI –6.21 to –1.73; mean difference [depression]: –1.34, 95% CI –2.63 to –0.04; mean difference [anxiety]: –1.46, 95% CI –2.7 to –0.23). Participants randomized to the intervention arm reported significantly higher well-being at 6 weeks than the waitlist control group (*F*_1,188_=12.38; *P*<.001; η_p_^2^=0.06; adjusted mean difference 3.78, 95% CI 1.66-5.9).

**Table 2:** Means and SDs for all measures at each follow-up point.

	Baseline, mean (SD)		6-week follow-up, mean (SD)		12-week follow-up, mean (SD)	
	RNT^a^-targeting app (n=119)	Waitlist control (n=117)	RNT-targeting app (n=95)	Waitlist control (n=96)	RNT-targeting app (n=73)	Waitlist control (n=73)
RRS^b^	51.08 (11.62)	51.51 (10.76)	49.91 (11.52)	52.88 (11.68)	47.86 (10.78)	49.3 (11.1)
PSWQ^c^	59.87 (10.82)	60.24 (10.4)	56.81 (9.38)	60.65 (11.08)	55.18 (10.49)	58.04 (11.08)
WEMWBS^d^	43.56 (7.92)	44.09 (7.08)	46.24 (8.95)	42.96 (7.47)	47.62 (6.96)	45.86 (7.13)
GAD-7^e^	9.40 (4.67)	9.04 (4.79)	7.97 (4.62)	9.14 (5.1)	7.45 (3.78)	7.90 (4.77)
PHQ-9^f^	8.61 (4.25)	8.56 (4.48)	8.57 (5.16)	9.73 (5.19)	7.85 (4.33)	8.49 (4.3)

^a^RNT: repetitive negative thinking.

^b^RRS: Ruminative Response Scale.

^c^PSWQ: Penn State Worry Questionnaire.

^d^WEMWBS: Warwick-Edinburgh Mental Well-being Scale.

^e^GAD-7: Generalized Anxiety Disorder-7.

^f^PHQ-9: Patient Health Questionnaire-9.

### Outcome at Follow-Up (12 Weeks)

At 12 weeks, participants who were initially allocated to use the RNT-targeting app maintained the changes that were made on all measures during the first 6 weeks, with no significant changes observed between the 2 follow-up points (see Table 3). After being introduced to the app at 6 weeks, participants in the waitlist control showed significant reductions in rumination, worry, anxiety, and depression and an increase in well-being (see Table 3). One-way ANCOVAs found no statistically significant differences between the 2 groups for any of the measures at the 12-week follow-up point (*F*_1, 143_≤1.17; *P*≥.28).

**Table 3:** Within-participants contrast between the 6- and 12-week follow-up point.

	RNT^a^-targeting app				Waitlist control		
	*F* test (*df*)	*P* value	Effect size (η_p_^2^)	*F* test (*df*)		*P* value	Effect size (η_p_^2^)
RRS^b^	2.63 (1, 144)	.11	0.02	22.09 (1, 144)		<.001	0.13
PSWQ^c^	1.47 (1, 144)	.23	0.01	12.2 (1, 144)		<.001	0.09
WEMWBS^d^	0.93 (1, 144)	.34	0.01	20 (1, 144)		<.001	0.12
GAD-7^e^	1.12 (1, 144)	.30	0.01	9.8 (1, 144)		.002	0.07
PHQ-9^f^	2.82 (1, 144)	.10	0.02	9.8 (1, 144)		.002	0.07

^a^RNT: repetitive negative thinking.

^b^RRS: Ruminative Response Scale.

^c^PSWQ: Penn State Worry Questionnaire.

^d^WEMWBS: Warwick-Edinburgh Mental Well-being Scale.

^e^GAD-7: Generalized Anxiety Disorder-7.

^f^PHQ-9: Patient Health Questionnaire-9.

### Adherence

Overall, 97 out of 119 (81.5%) participants in the intervention arm and 53 out of 96 (55.2%) in the waitlist control signed into the app. To estimate app usage, we calculated the mean number of days participants completed a daily mood rating (including those who never signed into the app). For those randomly allocated to use the RNT-targeting app, this was 8 (SD 10.92; range 0-40) days during the first 6-week period, and for the waitlist control, this was 3 (SD 6.31; range 0-31) days once given access to the app.

### UX Findings

When identifying positive attributes about the app, participants consistently highlighted the mood tracker and tool features as being beneficial. Participants also identified that the app provided them with useful skills to help improve their emotional management such as identifying unhelpful patterns, reflection, and analyzing their emotions more critically. Several participants noted that the app helped them feel relaxed and that it was most useful when they were experiencing heightened levels of distress or going through “a dark time.” When identifying areas to improve, several features appeared to affect participants’ motivation to regularly engage with it. A total of 9 comments highlighted that the app sent too many notifications which became very annoying; 11 comments alluded to the app being difficult to navigate and quite tiring to engage with on a regular basis; 8 comments noted experiencing frequent bugs and crashes; and 26 comments noted some design issues such as the range of emotions in the app being too limited, the questions used to evaluate daily events being repetitive, and the user interface being “too clinical” and not aesthetically pleasing. Overall, the average satisfaction rating from all participants who provided ratings regarding their experience (n=137) was 3.74 out of 5 (SD 0.73; ease of use: mean 3.99, SD 0.9; look and feel: mean 3.45, SD 1.08; features and functionality: mean 3.72, SD 0.87; content: mean 3.8, SD 0.94).

## Discussion

### Principal Findings

This was a prevention-mechanism trial that aimed to test whether an RNT-targeting self-help mobile phone app (MyMoodCoach) was effective at reducing worry and rumination in young adults. A further objective was to pilot the efficacy of the app in reducing symptoms of anxiety and depression and improving well-being.

As hypothesized, participants randomized to the MyMoodCoach app showed a significant decrease in both worry and rumination, relative to participants in the waitlist control condition. As hypothesized, participants randomized to the app also showed significant increases in well-being and reduction in anxiety and depression relative to the waitlist control group. All these changes were sustained at the 12-week follow-up point suggesting that any benefit of using the app is likely to persist over several months. Moreover, once they were given access to the app, participants in the waitlist control showed similar changes in RNT, anxiety, depression, and well-being, paralleling the benefits observed in the initial active intervention arm.

Patterns of change across the trial indicate that symptoms of depression during the first 6 weeks stayed constant in the intervention arm while worsening in the waitlist control group. This suggests that engaging in an intervention designed to target RNT may have a preventive effect on depressive symptoms consistent with the wider literature on rumination and worry as vulnerability factors for depression [39,71,72]. Further, this is consistent with previous RCTs that found that targeting RNT can have a medium-term preventive effect reducing the onset of depression [48,49]. The reduction of both anxiety and depression from a self-help app that explicitly targeted worry and rumination is also consistent with theoretical models proposing that RNT is a transdiagnostic risk factor that can affect symptoms associated with both disorders [41]. This research thus provides proof-of-principle of the value of targeting RNT as a mechanism to prevent anxiety and depression, consistent with the prevention mechanism trial approach. However, because we did not explicitly assess new onsets of major depression or anxiety disorders, and the follow-up was only short-term, longer-term follow-ups with an assessment of incidence are needed to rigorously test the potential of the self-help app targeting RNT as a prevention intervention. Despite app usage only being modest, it still had an effect. Further, the percentage of those not using the app (14%) was below the mean percentage of “non-users” reported in a recent meta-analytic review of adherence in mobile interventions (41.2%) [73].

In addition to its potential as a prevention intervention, the observed benefits of the app suggest there may be several clinical applications that could help reduce the burden on mental health services. As the intervention is focused on worry and rumination, the app could be offered to people who do not meet the severity threshold to access treatment for anxiety or depression, but still show high levels of RNT. It could also be offered as an interim approach for people on a waitlist for treatment as using the app may either reduce their associated symptoms or prevent their symptoms from getting worse. Moreover, as the app targets associated risk factors rather than a specific mental health problem, it may also help reduce the impact of stigma, which can prevent people from seeking support when distressed, and potentially increase engagement [74].

The study had several limitations. First, as all screening questionnaires were based on self-report, completed remotely, and no diagnostic interview was conducted, we cannot be sure that participants did not have a disorder at baseline. This also meant that we could not directly ascertain the effect of the app on the incidence of episodes of anxiety disorders and major depression. Second, participants were mainly White, female students, which may limit the generalizability of the findings, although we note a much greater proportion of females to males in the sample was expected given our inclusion criteria because elevated RNT tends to be much more common in females than males and has been found to partially account for the increased rates (nearly double) of depression and anxiety reported in females relative to males [75,76]. As such, this gender imbalance is not necessarily problematic for the purposes of developing an RNT-targeting preventative intervention for anxiety and depression but rather it accurately reflects the distribution of RNT in the target population. Future studies, however, will need to encourage more participation from other gender identities to confirm whether the app is effective for those who do not identify as female. Third, while the follow-up period in this study was appropriate for a prevention mechanism trial, a longer-term follow-up is needed to test the effect of the RNT-targeting app on preventing the incidence of episodes of major depression and generalized anxiety.

### Conclusions

Despite some limitations, the MyMoodCoach app has the potential to offer a valuable contribution toward large-scale effective prevention for young people [77]. These results provide proof of principle that the intervention can effectively target worry and rumination as possible prevention mechanisms for anxiety and depression in young people. As RNT is a well-established vulnerability factor, it is likely that this intervention will have a positive impact on the incidence of anxiety and depression in the medium-term.

## Appendix

Multimedia Appendix 1 Completed CONSORT-EHEALTH (Consolidated Standards of Reporting Trials of Electronic and Mobile HEalth Applications and on Line Tele Health) form.

Multimedia Appendix 2 Additional information about the RNT-targeting app.

## Abbreviations

ANCOVA: analysis of covariance
CONSORT: Consolidated Standards of Reporting Trials
CONSORT-EHEALTH: Consolidated Standards of Reporting Trials of Electronic and Mobile HEalth Applications and on Line Tele Health
ECoWeB: Assessing and Enhancing Emotional Competence for Well-being in the Young project
GAD-7: Generalized Anxiety Disorder questionnaire
MCID: minimum clinically important difference
PHQ-9: Patient Health Questionnaire-9
PSWQ: Penn State Worry Questionnaire
RCT: randomized controlled trial
RNT: repetitive negative thinking
RRS: Ruminative Response Scale
TAM: technology acceptance model
UX: user experience
WEMWBS: Warwick-Edinburgh Mental Well-being Scale
