# Supplementary material for: Evaluating the Effects of a Self-Help Mobile Phone App on Worry and Rumination Experienced by Young Adults: Randomized Controlled Trial
Source: JMIR Mhealth Uhealth. 2024 Aug 13;12:e51932. doi: 10.2196/51932 (PMC11350318; doi:10.2196/51932)
Supplement: Multimedia Appendix 2 [file mhealth_v12i1e51932_app2.docx]

**Overview of the MyMoodCoach App**

The app was designed for iOS and Android use. The app included text, pictures, audio-recordings, animations, audio-exercises to practice, questionnaires with tailored feedback, and quizzes, a self-monitoring feature with a regular daily mood rating, an emotion diary, and ecological momentary assessment (EMA) (MoodTracker) for more detailed analysis of mood, activity, and situational context, given evidence that this may increase engagement.

The app featured a menu structure including a dashboard to monitor notifications and progress, a library function that had psychoeducation and explanatory animated videos, and an explore function to graph the self-monitoring responses made by the participant. The app also included “Challenges” that provide learning exercises (for example, behavioral exercises), and “Tools” that are brief strategies young people can use in the moment when they need them (for example, compassion and relaxation exercises).

The Rumination module focused on improving emotional regulation by reducing maladaptive worry and rumination, well-established risk factors for poor mental health and increasing constructive alternatives and problem-solving. It adapted a proven intervention, which used proven cognitive-behavioural therapy principles and included identifying warning signs for worry, repeated practice to train out of unhelpful habits and build helpful habits, and the training of useful alternative strategies such as being more specific, relaxation, problem-solving, and self-compassion. This intervention is effective in reducing and preventing depression and anxiety in face-to-face therapy and in web-based interventions for young adults including an entirely self-help variant. Further details and information are given in the tables below.

**Table S1.** Description of selection of Tools within the MyMoodCoach self-help RNT app.

| **Name of Tool** | **Description of Feature** |
| --- | --- |
| Take a breath | Animated video illustrating how to practice slow breathing. |
| Count your blessings | Participant identifies and writes down three good things that happened that day and what caused the good things to happen |
| Be kind to yourself | Animated video explaining the value of increasing self-compassion to tackle stress and rumination. Active strategy to implement kind self-talk in a current situation or from a memory which is worrying them. Participant identifies the scenario, and what their inner critic is saying. Prompts provided as to more supportive things to say to self, including via an audio-exercise. Prompt to encourage positive self-talk which can be used in that moment and applied in the future. |
| Opposite action | Animated video explaining the value of acting opposite to negative emotion. Participant identifies a current emotion and works through multiple-choice questions which give direct feedback on how they can act in an opposite way to their current emotion in terms of posture, facial expression and action. |
| Slow things down | Questions to help user to focus on what is happening in the moment, identify one task to focus on and prioritise and to create manageable steps to proceed with the task. Focused on making immediate behavioural and attentional changes. |
| Be specific | Animated video explaining the difference between abstract versus concrete thinking and how the latter is more adaptive. Participant chooses a problem of their own and works through exercise either vis audio-recording or through text prompts to consider the problem in a detailed, specific, concrete and contextualised way and to break it down into realistic and manageable actions. |
| Relaxation | Psychoeducation on usefulness of relaxation. 3 audio exercises to use to relax in the moment reflecting progressive muscular relaxation, focus on present and body scan exercises. |

**Table S2.** Description of selection of Challenges within the MyMoodCoach self-help RNT app.

| **Name of Challenge** | **Description of Feature** |
| --- | --- |
| Identifying warning signs | Animated video explaining how to break the worry/rumination habit cycle by spotting and removing triggers. Exercise involving multiple choice questions of common triggers and warning signs including thoughts, bodily changes, situations, emotions, and behaviours to identify the relevant warning signs for worry and rumination for that individual. Contingent feedback on relevant strategies and challenges/tools in the app provided in response to answers. |
| Experience Kind and Unkind Talk | Animated video explaining the value of increasing self-compassion to tackle stress and rumination. Behavioural experiment comparing the differences the user notices between self-critical versus kind self-talk: Participants asked to think of various scenarios and to identify their inner voice and compare talking to themselves with inner critic versus more kindly voice. Prompts are given to identify their thoughts and suggestions on how to develop a compassionate inner voice |
| Experience abstract and concrete thinking | Animated video providing psychoeducation on abstract and concrete and helpful and unhelpful thinking styles. Experiential exercise/behavioural experiment consisting of audio-exercises in which user visualise sitting in a café waiting for someone significant and that person is late with questions prompting respectively abstract versus concrete thinking styles. Reflection on thoughts and feelings experienced when thinking in abstract and then concrete thinking style, and identify most helpful specific thinking. |

Example screenshots illustrating relevant screens that users see in the various forms of the app are shown below.


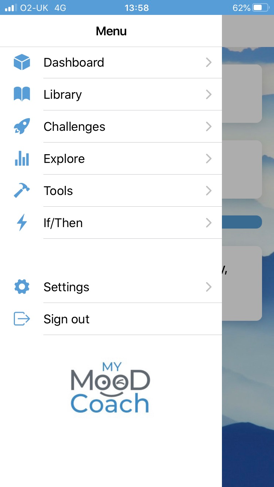


**Figure S1.** Illustration of App menu.


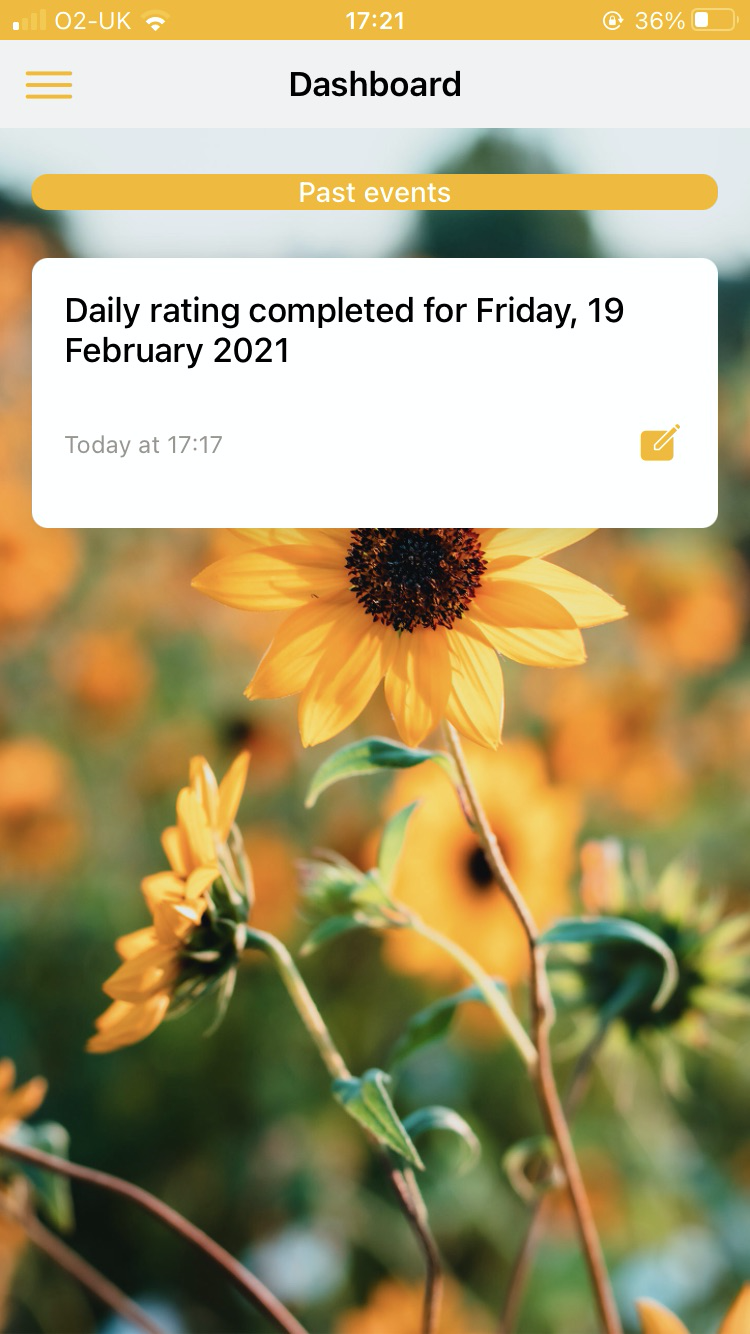


**Figure S2.** Dashboard on app indicating daily rating completed.


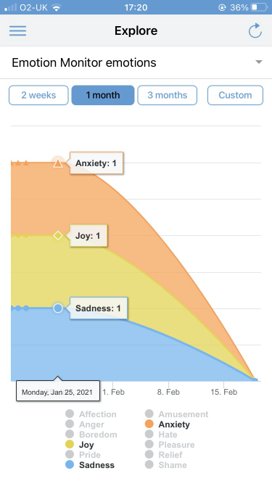

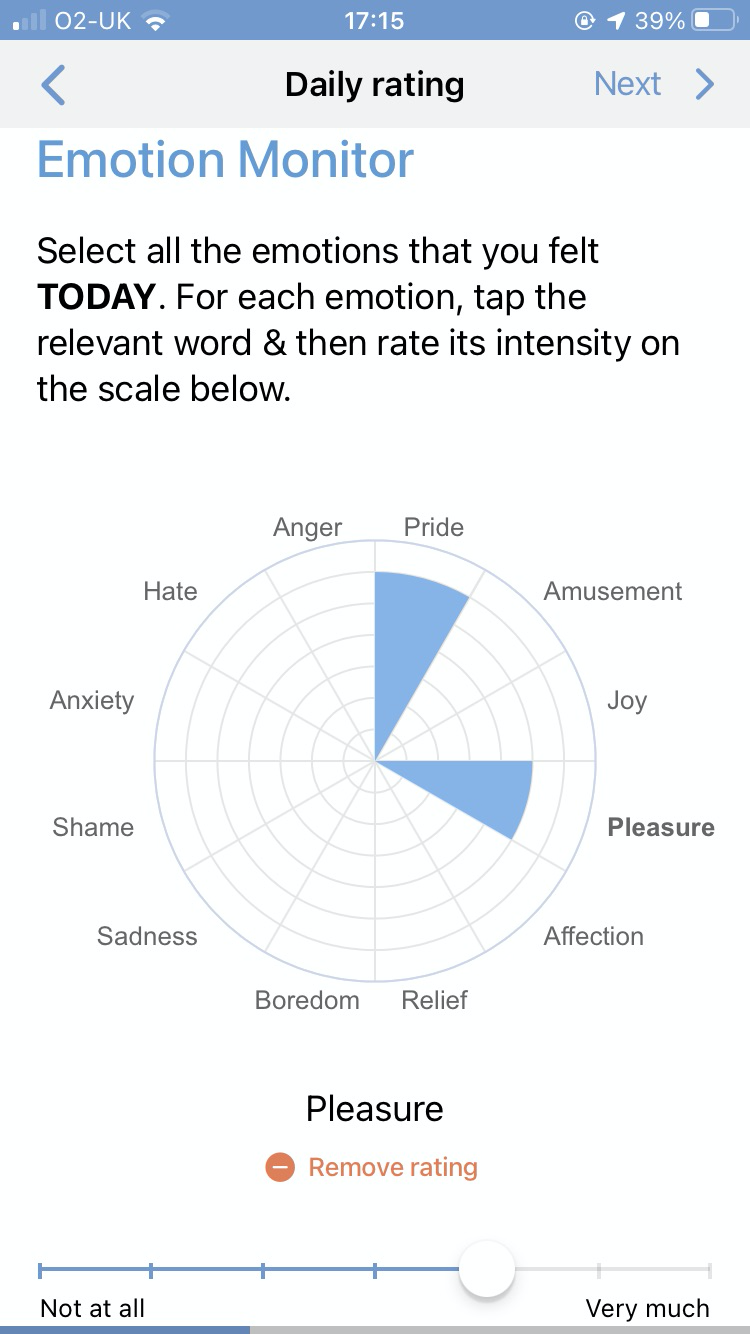


**Figure S3.** Emotion wheel used to rate emotion in emotion monitor (once daily), Ecological momentary assessment and emotion diary.


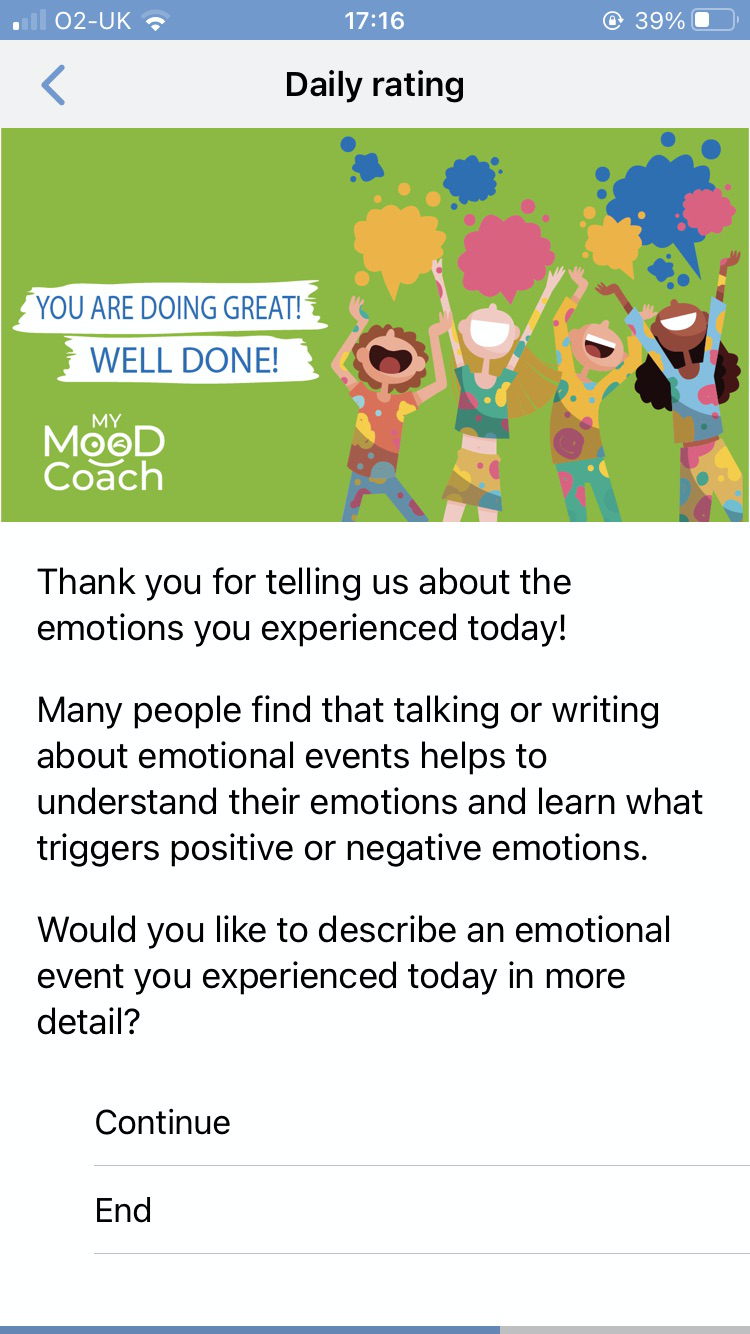


**Figure S4.** Feedback screen after completing Emotion Monitor.


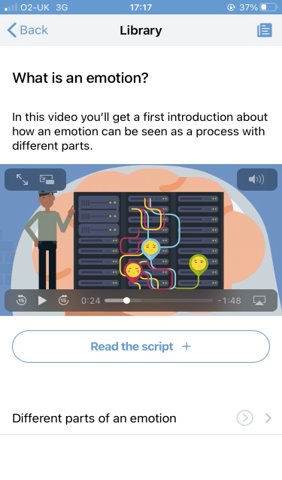


**Figure S5.** Library of visual resources (what is an emotion?).
